# Effect of having floats on cardiorespiratory response during eggbeater kick

**DOI:** 10.1186/2046-7648-4-S1-A50

**Published:** 2015-09-14

**Authors:** Yosuke Sasaki, Hideki Takagi, Bun Tsuji, Yosuke Murase, Shozo Tsubakimoto, Takeshi Nishiyasu

**Affiliations:** 1 Graduate School of Comprehensive Human Sciences, University of Tsukuba, Tsukuba, Japan; 2 Institute of Health and Sport Sciences, University of Tsukuba, Tsukuba, Japan; 3 Faculty of Human Development, Kobe University, Kobe, Japan

## Introduction

The eggbeater kick is used to tread water during emergency situations (*e.g*. boat capsize) and becomes necessary skill for those without a self-floatation device to prevent primary and secondary drowning. Utilizing floating debris or objects to minimize the metabolic cost of performing this technique is essential during such emergencies. However, no study has investigated the effects of having floats on the physiological demands of performing the eggbeater kick, and quantifying these effects forms the focus of the current experiment.

## Methods

Twelve males, each trained in performing the eggbeater kick, treaded water using this technique for five consecutive, 3 min periods, with each period varying only in the quantity of floatation used (No-, One-, Two-, Three- and Four-floats). Floats were 500 mL plastic bottles filled with air, each equal to ~0.6 kgf of buoyancy. Subjects were instructed to keep their head above water level for the duration of each exercise period, with fitting a brief swimsuit, in an indoor swimming pool containing chlorinated water (depth and mean (SD) temperature of water: 2 m and 26.7 (0.4) °C, respectively). Oxygen consumption, heart rate, ratings of perceived exertion and the difficulty of breathing index were measured. Data were sampled during the final minute of each exercise period and used for subsequent analyses.

## Results

Oxygen consumption and heart rate during exercise with additional floatation were significantly less than during exercise without floats (oxygen consumption: 1.80 (0.31) L.min^-1^; heart rate: 113 (13) bpm), and decreased in proportion to the number of floats used (*r *= 0.99; p<0.05). This was equivalent to a decrease in oxygen consumption and heart rate of 0.17 L.min^-1 ^and 4 bpm per one-float, or 0.29 L.min^-1 ^and 8 bpm per one-kgf of buoyancy (p<0.05). In addition, ratings of perceived exertion and difficulty of breathing scores were generally less when additional floats were used (p<0.05); these scores also demonstrated strong linear relationships with the number of floats used (*r *= 0.99; p<0.05).

## Discussion

A previous study reported that trapping air between clothing layers increased the buoyancy just after immersion (1). Similarly, having floats might create the additional buoyancy during eggbeater kick in present study. Further, the buoyancy might reduce the vertical force required to keep the head above the water surface, and potentially prolong the duration one can tread water in survival situations, particularly in the absence of a life jacket. To consider more scenarios where accidental immersion occur in some populations, further investigations may be required in the future (*e.g*. effects of water temperature, salinity, viscosity and currents, wearing clothing, inherent buoyancy and performing levels of subjects on the cardiorespiratory responses during eggbeater kick).

## Conclusion

These data indicate that additional floatation, even as little as one empty water bottle, can significantly reduce the physiological demands of treading water using the eggbeater kick, and increase survival time during emergency scenarios. The importance of obtaining additional floatation in the event of an accidental water immersion should be included in water survival training and safety protocols.
